# Assessing the reliability of non-cycloplegic refraction in children: a machine learning approach based on non-cycloplegic parameters

**DOI:** 10.3389/fpubh.2026.1822514

**Published:** 2026-06-29

**Authors:** Haoqiang Cui, Jianning Huang, Zhenbao Zhou, Yuyang Yang, Bowen Zhang, Xiaobin Hong, Jinyu Zhong, Kunhong Xiao, Jiahao Liu, Huiying Rao, Li Li, Junhua Zhang

**Affiliations:** 1 Department of Ophthalmology and Optometry, Fujian Medical University, Fuzhou, China; 2 Department of Ophthalmology, Shengli Clinical Medical College of Fujian Medical University, Fuzhou University Affiliated Provincial Hospital, Fuzhou, China; 3 Fuzhou Ruiming Aier Eye Clinic, Fuzhou, China; 4 Department of Ophthalmology, The First Affiliated Hospital of Xiamen University, Xiamen, China; 5 Centre for Eye Research Australia, Royal Victorian Eye and Ear Hospital, East Melbourne, VIC, Australia; 6 Ophthalmology, Department of Surgery, The University of Melbourne, Melbourne, VIC, Australia

**Keywords:** accommodative function, cycloplegic refraction, logistic regression, machine learning, nomogram, refractive error

## Abstract

**Background:**

Traditional cycloplegic refraction is the gold standard for pediatric vision screening but is often limited by low efficiency and poor compliance. This study aimed to develop a machine learning model using non-cycloplegic visual function and refractive parameters to evaluate the reliability of non-cycloplegic refraction in children and adolescents. The model aims to identify individuals at risk of significant refractive error deviation (defined as an absolute difference in spherical equivalent [DSE] > 0.25 D) and to determine the necessity for cycloplegic intervention.

**Methods:**

A total of 300 children and adolescents (547 eyes) were included. Eighteen demographic, refractive, and binocular vision variables were collected. Feature selection was performed using the univariate logistic regression and least absolute shrinkage and selection operator (LASSO). Five models (decision tree, logistic regression, support vector machine, random forest, and multilayer perceptron) were developed. Performance was evaluated using accuracy, sensitivity, specificity, area under the receiver operating characteristic curve (AUC), and decision curve analysis (DCA), and interpretability was assessed using SHapley Additive exPlanations (SHAP).

**Results:**

An absolute DSE greater than 0.25 D was associated with multiple accommodative and refractive parameters. Logistic regression showed the best performance, with an AUC of 0.871 (95% CI: 0.798–0.944), an accuracy of 0.809, a sensitivity of 0.771, and a specificity of 0.827. The most important predictors included the monocular estimation method (MEM), negative relative accommodation (NRA), positive relative accommodation (PRA), cylindrical, and accommodative facility. A nomogram was constructed to estimate the probability of DSE greater than 0.25 D.

**Conclusion:**

Machine learning models based on routine non-cycloplegic parameters can effectively identify children who require cycloplegic refraction, providing an interpretable and practical decision-support tool to reduce unnecessary cycloplegia and improve clinical efficiency.

## 1 Introduction

Refractive error is one of the most common visual problems among children and adolescents (1). This developmental period represents not only a critical stage for visual system maturation but also a phase of markedly increased visual demand and the highest risk for the onset and progression of myopia (2). Refractive error, including myopia, hyperopia, and astigmatism, is therefore a key determinant of visual health in this population. Accurate assessment of refractive status is essential for myopia control, refractive screening, and clinical decision-making.

In clinical practice, owing to the strong accommodative ability and accommodative tension in children and adolescents, the spherical equivalent (SE) often differs substantially before and after cycloplegia (3–5). This discrepancy can lead to underestimation of true hyperopia due to masked latent hyperopia, and overestimation of myopia due to accommodative spasm or pseudomyopia. Such uncertainty in SE poses major challenges for accurate spectacle prescription and for the formulation of appropriate myopia control strategies. Consequently, cycloplegic refraction remains the gold standard for determining true refractive status (6–8).

However, cycloplegic refraction requires the instillation of mydriatic and cycloplegic agents, which presents multiple barriers and challenges for children and their caregivers. First, although uncommon, drug allergy is an absolute contraindication and cannot be ignored, as some children are unable to tolerate or are allergic to cycloplegic agents (9, 10). Second, even in the absence of allergy, cycloplegia-induced near blur and photophobia significantly interfere with daily activities such as studying and recreation, often resulting in reluctance to undergo repeat examinations (11, 12). In addition, a small proportion of children may experience ocular redness, eye pain, or headache after cycloplegia. More importantly, in school-based screenings, primary care settings, and large-scale epidemiological surveys, the widespread use of cycloplegia is constrained by manpower, time, drug availability, and parental acceptance. Therefore, there is a pressing clinical need for a non-invasive and efficient method to identify individuals with significant accommodative tension or refractive variability, namely those in whom the difference between cycloplegic SE and non-cycloplegic measurements exceeds a clinically meaningful threshold (e.g., >0.25 D).

In recent years, the rapid advancement of artificial intelligence (AI) has revolutionized the field of ophthalmology. AI-driven screening and grading systems have demonstrated exceptional performance in managing various ocular conditions, including age-related macular degeneration, diabetic macular edema, and dry eye disease. By significantly enhancing diagnostic accuracy and streamlining clinical workflows, these technologies provide robust support for disease management (13–16). Furthermore, the integration of AI has demonstrated significant potential in certain specialized fields, such as predicting orthokeratology lens decentration and driving the digital transformation of optometric education (e.g., virtual simulation for contact lens fitting) (17, 18). In the field of refractive error, research efforts have increasingly shifted from “instrument-based improvement” to “data-driven predictive modeling” to enhance the clinical utility of non-cycloplegic refraction. Several international studies have attempted to develop deep learning or machine learning models using multimodal inputs, such as age, uncorrected visual acuity, axial length (AL), corneal curvature, lens diopter, and retinal images, to predict cycloplegic SE or to determine whether non-cycloplegic refraction can substitute for cycloplegic measurements (19–24). However, most existing studies focus on predicting the post-cycloplegic refractive value itself rather than directly addressing the clinical decision of whether cycloplegia is necessary, and their predictive accuracy remains limited. Clinically, the key determinant for cycloplegia is whether the difference in spherical equivalent before and after cycloplegia (DSE) reaches a clinically meaningful threshold (e.g., 0.25 D). Although some studies have attempted to predict the need for cycloplegia using machine learning, they have largely relied on basic refractive parameters and have not sufficiently incorporated critical indicators of accommodative function.

Therefore, this study aims to integrate demographic characteristics, accommodative function, binocular vision parameters, and autorefraction-derived variables as candidate predictors, with the absolute DSE greater than 0.25 D serving as the clinically relevant outcome. By developing and validating a multivariable predictive model, we seek to provide a practical clinical tool to efficiently identify children and adolescents who truly require cycloplegic refraction, thereby optimizing clinical workflows, reducing patient burden, conserving medical resources, and enabling more precise and patient-centered refractive assessment.

## 2 Methods

### 2.1 Study participants

This retrospective study included children and adolescents who attended Fuzhou Aier Eye Hospital between October 2024 and October 2025 and underwent non-cycloplegic refraction, cycloplegic refraction, visual function testing, corneal measurements, and ocular biometry. A total of 300 participants were initially screened. Based on data completeness and the study outcome, 547 eyes were finally included for model development to predict whether the absolute DSE exceeded 0.25 D. All participants had complete pre-cycloplegic refractive and visual function data, including autorefraction and relevant visual function parameters, and underwent standardized post-cycloplegic retinoscopy during the same visit. None of the enrolled subjects had ocular diseases that could affect the accuracy of refractive measurements. Exclusion criteria included a history of corneal scarring, congenital cataract, keratoconus, or other media opacities that might interfere with refractive assessment, as well as incomplete clinical data. To ensure measurement consistency and comparability, all cycloplegic and refractive examinations were performed by experienced optometrists following a standardized protocol, and the autorefraction values were recorded as the mean of three repeated measurements.

This study was approved by the Institutional Review Board (approval number: K2025-11-001), and written informed consent was obtained from the guardians of all participants. All procedures adhered to the principles of the Declaration of Helsinki.

### 2.2 Data collection

Demographic and clinical data were collected, including age, sex, and baseline best-corrected visual acuity (BCVA). All participants completed standardized visual function and refractive examinations before cycloplegia. Refractive parameters included sphere, cylinder, and SE obtained from non-cycloplegic autorefraction, as well as corneal curvature K1, K2, mean keratometry (K), and corneal astigmatism. Additional ocular parameters included AL and intraocular pressure (IOP) measured by non-contact tonometry. Accommodative and binocular visual function measures included negative relative accommodation (NRA), positive relative accommodation (PRA), accommodative response assessed by monocular estimation method (MEM) retinoscopy, accommodative amplitude (AMP), near point of convergence (NPC), and accommodative facility, which was recorded as whether the participant could successfully complete the +2.00 D flipper test.

### 2.3 Feature selection

To construct an efficient and robust predictive model, a two-pronged, independent feature selection strategy was simultaneously implemented to cross-validate the 18 candidate variables from both univariate and multivariate perspectives. On one path, univariate logistic regression analysis was conducted to identify variables significantly associated with the absolute DSE. Concurrently and independently, a least absolute shrinkage and selection operator (LASSO) regression model with L1 regularization was constructed for multivariate feature shrinkage. Within the LASSO framework, 10-fold cross-validation based on the ‘one-standard-error rule’ (*λ*.1se) was applied to determine the optimal penalty parameter λ, thereby generating a parsimonious feature set and mitigating overfitting risks by shrinking non-informative coefficients strictly to zero. Finally, to eliminate potential selection biases inherent to any single approach, the intersection of candidate predictors independently yielded by both parallel methods was defined as the finalized feature set for downstream machine learning modeling.

### 2.4 Model development and performance evaluation

Decision tree (DT), logistic regression (LR), support vector machine (SVM), multilayer perceptron (MLP), and random forest (RF) algorithms were used to construct predictive models. The dataset was randomly split into a training set (80%) and a testing set (20%). To avoid potential data leakage caused by bilateral eye inclusion, eyes from the same participant were assigned to the same dataset partition. During model training, 10-fold cross-validation and grid search were applied for hyperparameter optimization to achieve optimal performance.

To comprehensively evaluate the ability of each model to predict DSE status, accuracy, sensitivity, specificity, area under the receiver operating characteristic curve (AUC), and decision curve analysis (DCA) were calculated in the testing set. The best-performing model was selected for further interpretation, and a clinical prediction tool was developed based on this model. Feature importance analysis and SHapley Additive exPlanations (SHAP) values were used to improve model transparency and interpretability. In addition to the primary analysis using an absolute DSE threshold of >0.25 D, sensitivity analyses were performed using alternative thresholds of >0.50 D and >0.75 D to evaluate the robustness of the final logistic regression model across different levels of agreement between non-cycloplegic and cycloplegic refraction.

### 2.5 Statistical analysis

All continuous variables were first assessed for normality using the Shapiro–Wilk test. Because all *p* values were less than 0.05, the variables were considered non-normally distributed and are presented as median and interquartile range (P25, P75), with group comparisons performed using the Mann–Whitney U test. Categorical variables are presented as frequencies and percentages and were compared using the chi-square test. Modeling was conducted using the tidymodels framework in R. To enhance model interpretability, SHAP values were calculated using the shapviz package to quantify the contribution of each variable to the prediction, and SHAP summary and dependence plots were generated to identify key predictors. For clinical application, a nomogram was developed based on the logistic regression model using the rms package. Model fitting was performed with the lrm() function, and the nomogram was generated using the nomogram() function to visualize key predictors and generate individualized risk scores. Because both eyes from some participants were included in the primary analysis, additional sensitivity analyses were performed to account for potential inter-eye correlation. Generalized estimating equations (GEE) with participant-level clustering and an exchangeable correlation structure were applied for the final logistic regression model. In addition, a single-eye sensitivity analysis was conducted by randomly selecting one eye from each participant, and the predictive performance of the logistic regression model was re-evaluated in the resulting dataset. All statistical analyses were performed using R software version 4.5.0.

## 3 Results

### 3.1 Baseline characteristics

When an absolute DSE greater than 0.25 D was used as the outcome, significant differences were observed between participants with DSE > 0.25 D and those with DSE ≤ 0.25 D (Table 1). Compared with the DSE ≤ 0.25 D group, participants in the DSE > 0.25 D group had significantly lower cylindrical diopter, corneal astigmatism, PRA, and NRA values (all *p* < 0.05). In addition, these participants had significantly shorter AL (*p* < 0.05) and significantly higher corneal curvature values (K1, K2, and K) and AMP (all *p* < 0.05). Moreover, a significantly higher proportion of participants in the DSE > 0.25 D group failed the plus-lens stage of the accommodative facility test (*p* < 0.001).

**Table 1:** Baseline characteristics of the study population according to DSE > 0.25 D.

Variable	DSE ≤ 0.25D *N* = 208^1^	DSE > 0.25D *N* = 339^1^	*p*-value^2^
Sex			0.2
Female	102 (49%)	184 (54%)	
Male	106 (51%)	155 (46%)	
Flipper			<0.001
Pass (Plus-lens stage)	113 (54%)	72 (21%)	
Fail (Plus-lens stage)	95 (46%)	267 (79%)	
BCVA			0.050
BCVA<1.0	21 (10%)	19 (5.6%)	
BCVA≥1.0	187 (90%)	320 (94%)	
Cycloplegic_Sphere	−1.25 (−2.25, −0.50)	−0.50 (−1.75, 0.75)	<0.001
Cycloplegic_Cylinder	−0.75 (−1.50, −0.50)	−0.75 (−1.75, −0.25)	0.9
Cycloplegic_Spherical_Equivalent	−1.75 (−2.75, −0.88)	−0.88 (−2.13, 0.25)	<0.001
Cycloplegic_Axis	165 (11, 174)	165 (9, 175)	>0.9
Age	10 (8, 12)	10 (9, 12)	0.3
MEM	1.00 (0.75, 1.25)	1.00 (0.75, 1.00)	<0.001
NRA	2.25 (2.00, 2.50)	2.00 (1.75, 2.25)	<0.001
PRA	−2.00 (−2.75, −1.50)	−2.75 (−3.50, −2.00)	<0.001
AMP	13.20 (12.50, 13.90)	13.50 (12.50, 14.30)	0.007
NPC	6.00 (5.70, 7.00)	6.00 (5.70, 7.00)	0.084
Autorefraction S (non-cycloplegic)	−1.25 (−2.25, −0.50)	−1.25 (−2.50, 0.00)	0.14
Autorefraction C (non-cycloplegic)	−0.75 (−1.25, −0.25)	−1.00 (−1.75, −0.50)	0.013
Autorefraction SE (non-cycloplegic)	−1.81 (−2.81, −1.13)	−1.75 (−3.25, −0.63)	0.3
Autorefraction_K1	42.25 (41.50, 43.00)	42.50 (41.50, 43.50)	0.035
Autorefraction_K2	43.75 (43.00, 44.50)	44.25 (43.25, 45.25)	<0.001
Autorefraction_K	43.00 (42.25, 43.75)	43.50 (42.50, 44.50)	0.001
Autorefraction_Corneal_Astigmatism	−1.50 (−1.75, −1.00)	−1.50 (−2.25, −1.00)	0.003
IOP	15 (13, 17)	15 (13, 17)	0.022
AL	24.35 (23.83, 25.04)	23.96 (23.28, 24.81)	<0.001

^1^*n* (%); Median (Q1, Q3); ^2^Pearson’s Chi-squared test; Wilcoxon rank sum test.

### 3.2 Feature selection

**Figure 1:**
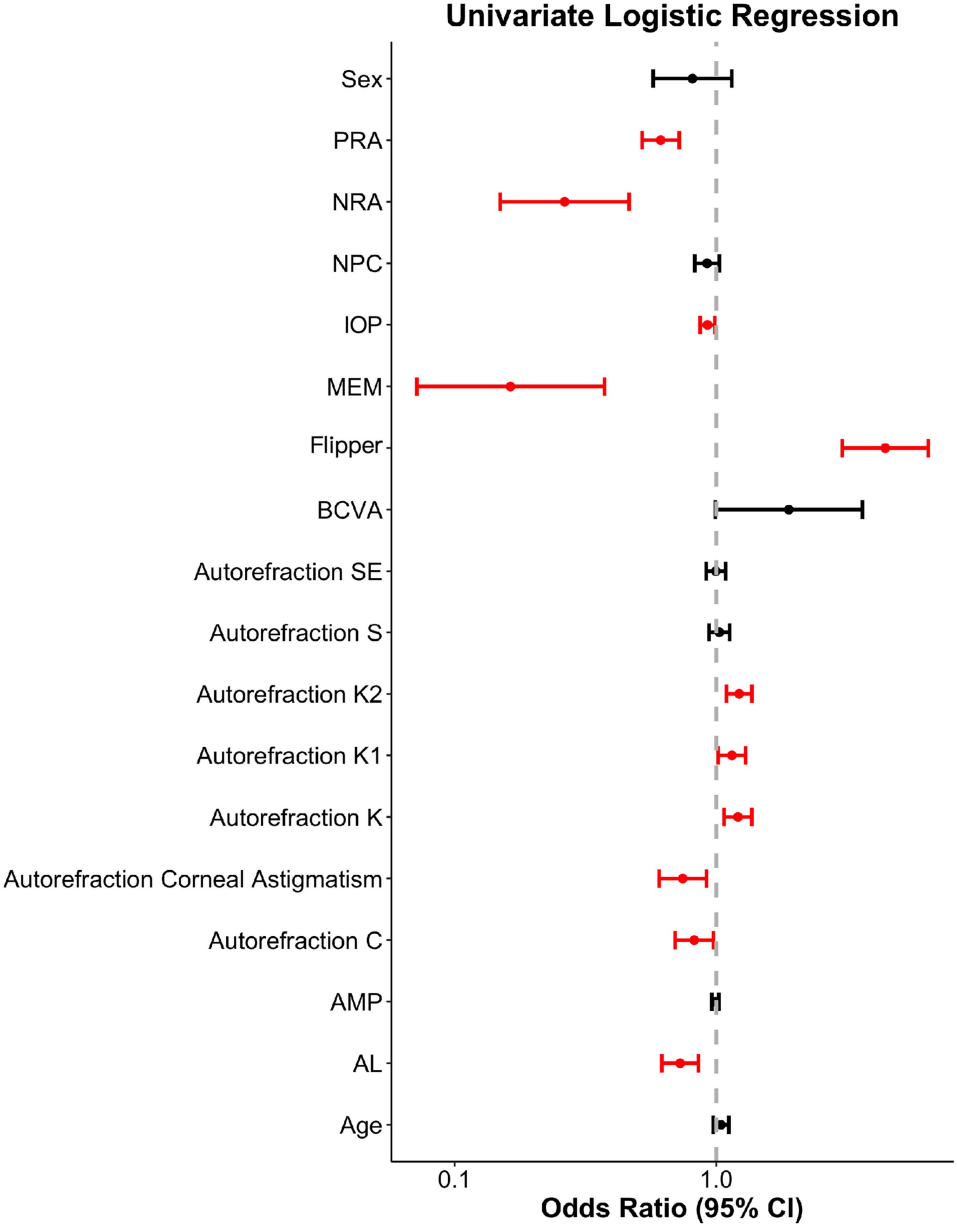
Significant variables identified by univariate logistic regression for the outcome of DSE > 0.25 D.

Among the 18 collected variables, univariate logistic regression analysis identified 11 independent factors associated with an absolute DSE greater than 0.25 D. Cylindrical diopter, NRA, IOP, PRA, MEM, AL, and corneal astigmatism were identified as negative predictors, whereas accommodative facility (passing the plus-lens stage) and corneal curvature parameters (K1, K2, and K) were identified as positive predictors of DSE > 0.25 D (Figure 1).

**Figure 2:**
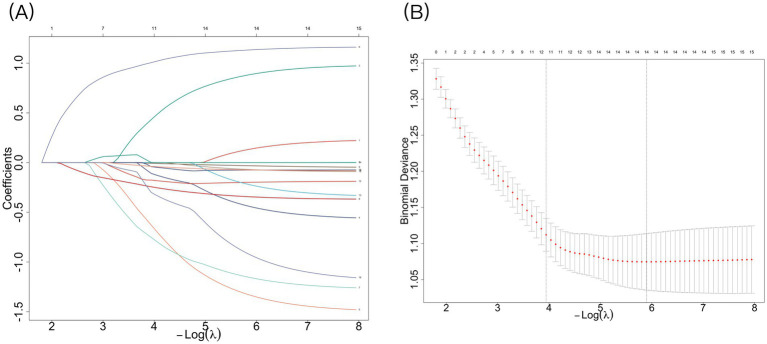
Feature selection using LASSO regression. **(A)** LASSO coefficient profiles of the 18 candidate predictors for the outcome of DSE > 0.25 D. **(B)** Ten-fold cross-validation was performed to identify the optimal penalty parameter (*λ*), and λ.1se was selected as the final tuning parameter for model development.

To further control for potential confounding factors affecting the prediction of DSE > 0.25 D, LASSO regression was applied for refined feature selection (Figure 2). By taking the intersection of variables selected by LASSO regression and univariate logistic regression, five predictors were ultimately identified from the initial 18 candidate variables: MEM, NRA, PRA, cylindrical diopter, and accommodative facility (passing the plus-lens stage).

Multicollinearity analysis of the selected predictors showed that all variance inflation factors (VIFs) were less than 2.5, indicating no significant multicollinearity among these variables (Table 2).

**Table 2:** Assessment of multicollinearity among predictors using variance inflation factors (VIFs).

Variable	VIF	*p*_value
MEM	1.059	0.006570
NRA	1.190	0.019300
Flipper	1.137	5.84e-11
PRA	1.290	0.000567
Autorefraction_C	1.009	0.000652

### 3.3 Model selection and performance evaluation

**Figure 3:**
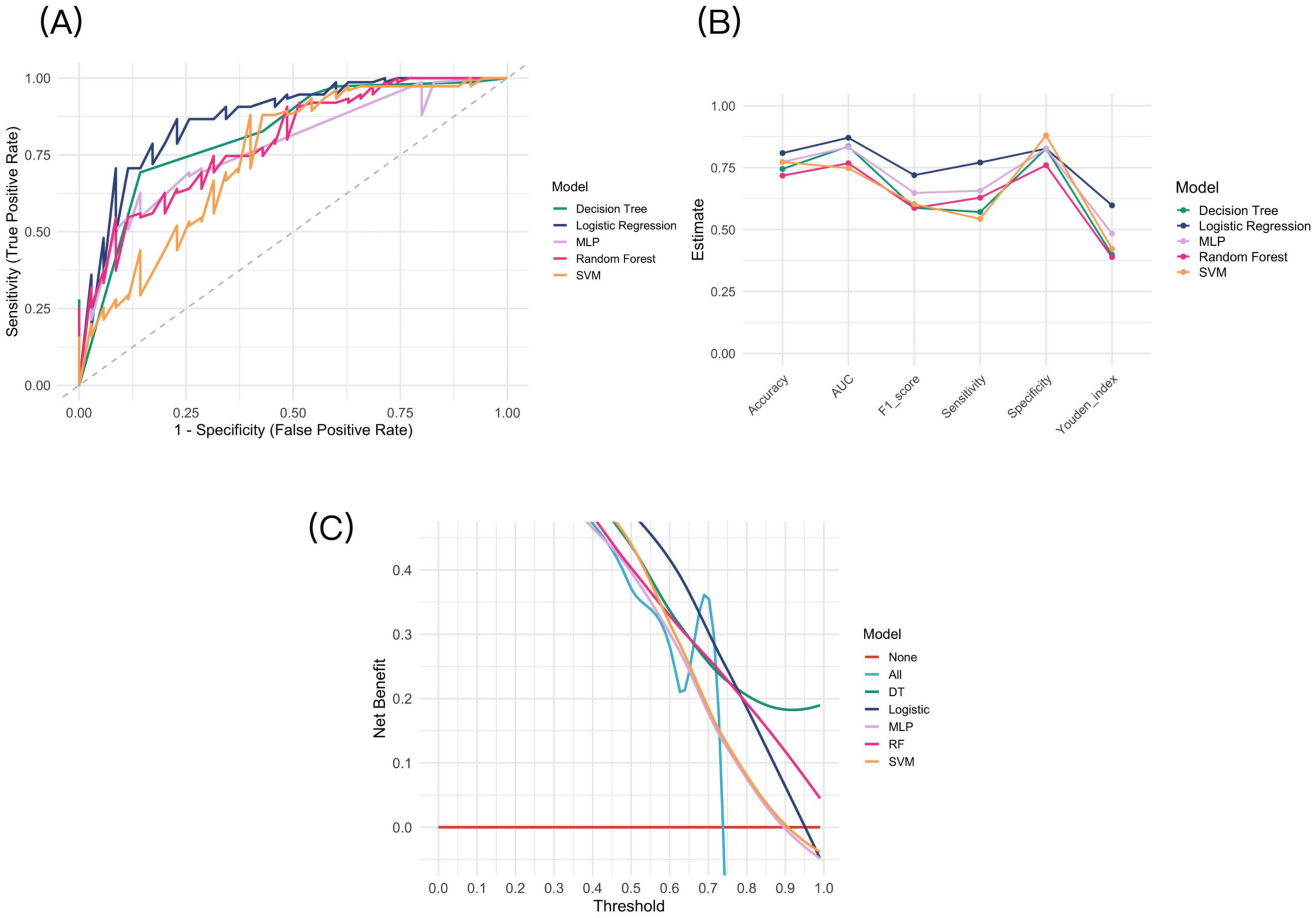
Performance comparison of five machine learning models for predicting DSE > 0.25 D. **(A)** ROC curves of the models for discriminating DSE > 0.25 D; **(B)** comparison of performance metrics of the five models; **(C)** DCA of the models for evaluating their net clinical benefit.

Based on the selected predictors, five machine learning models were developed: DT, LR, SVM, MLP, and RF. For predicting DSE > 0.25 D, the LR model demonstrated the best performance in the testing set, with an accuracy of 0.809, a sensitivity of 0.771, a specificity of 0.827, an F1 score of 0.720, an AUC of 0.871 (95% CI: 0.798–0.944), and a Youden index of 0.598 (Table 3; Figures 3A,B).

**Table 3:** Performance of 5 machine learning models for the prediction of DSE > 0.25 D.

Model	Accuracy	AUC (95% CI)	Sensitivity	Specificity	Youden index	F1
DT	0.745	0.836 (0.762, 0.910)	0.571	0.827	0.398	0.588
LR	0.809	0.871 (0.798, 0.944)	0.771	0.827	0.598	0.720
MLP	0.773	0.833 (0.745, 0.922)	0.657	0.827	0.484	0.648
RF	0.718	0.768 (0.674, 0.862)	0.629	0.760	0.389	0.587
SVM	0.773	0.748 (0.644, 0.852)	0.543	0.880	0.423	0.603

DCA showed that within a narrow threshold probability range of 0.67–0.72, the LR model provided a lower net clinical benefit than the treat-all strategy. However, across all other threshold ranges, the LR model yielded a higher net clinical benefit than both the treat-all and treat-none strategies (Figure 3C).

Sensitivity analyses using alternative DSE thresholds demonstrated relatively stable predictive performance of the final logistic regression model. When thresholds of DSE > 0.50 D and >0.75 D were applied, the model achieved AUCs of 0.835 (95% CI: 0.758–0.911) and 0.862 (95% CI: 0.792–0.932), respectively. Detailed performance metrics are presented in Supplementary Table 3.

### 3.4 SHAP interpretability analysis and nomogram construction

**Figure 4:**
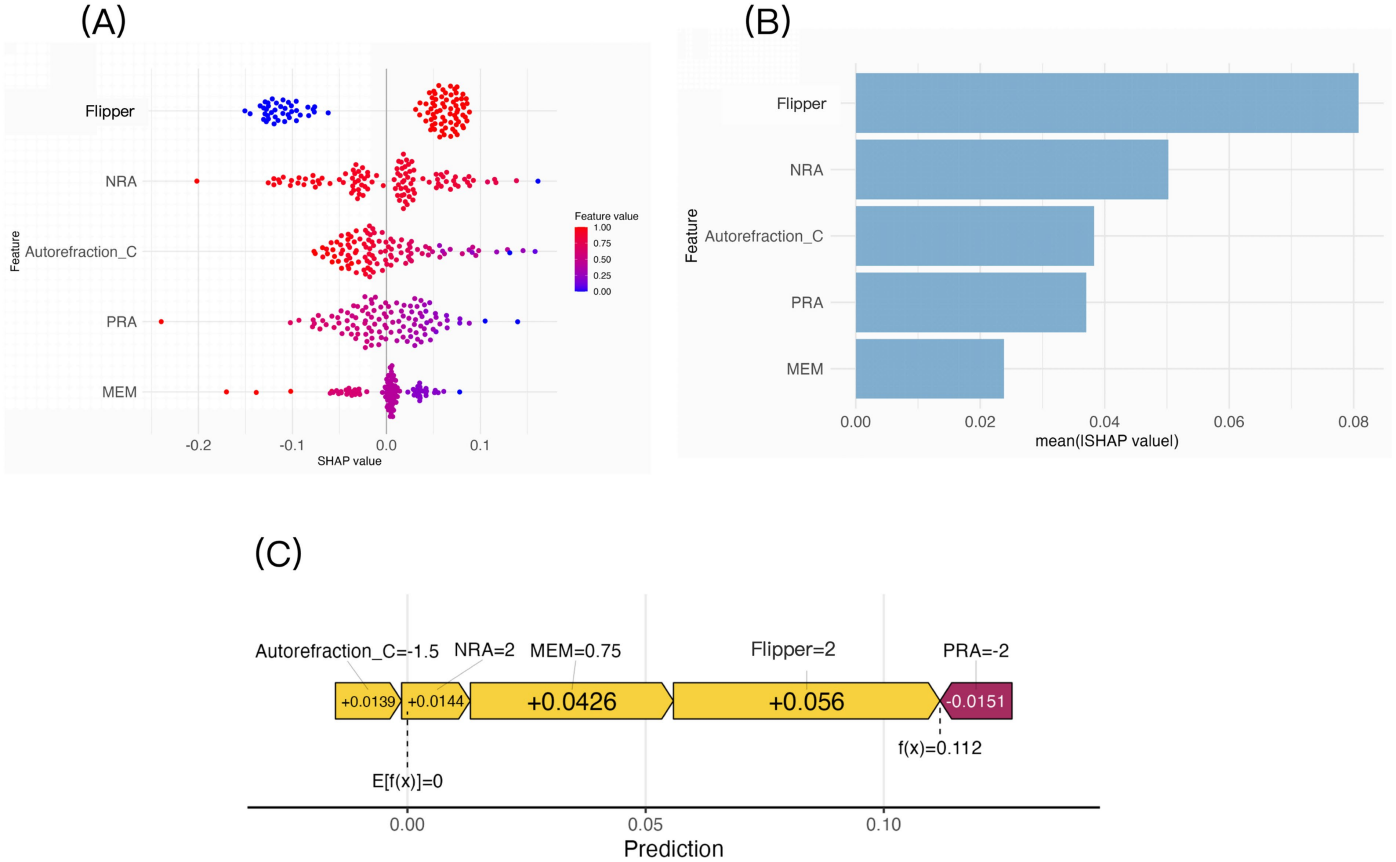
SHAP-based interpretation of the best-performing predictive model. **(A)** SHAP summary plot showing the overall contribution and direction of each feature to the prediction of DSE > 0.25 D across all samples; **(B)** SHAP bar plot ranking feature importance based on the mean absolute SHAP values; **(C)** SHAP force plot illustrating the prediction for a representative individual and the positive and negative contributions of each feature.

SHAP analysis showed that, for the prediction of DSE > 0.25 D, accommodative facility made a strong positive contribution to the model output, whereas MEM, PRA, NRA, and cylindrical diopter contributed negatively (Figure 4A). Feature importance ranking indicated that accommodative facility was the most influential predictor, followed by NRA, cylindrical diopter, PRA, and MEM (Figure 4B). The SHAP force plot illustrated individualized prediction explanations. For the representative case, failure in the accommodative facility test exerted a substantial positive impact on the predicted risk (SHAP = +0.056). In addition, an NRA of +2.00 D (SHAP = +0.014), a cylindrical diopter of −1.50 D (SHAP = +0.014), and an MEM of +0.75 D (SHAP = +0.043) all contributed positively to the prediction, whereas a PRA of −2.00 D contributed negatively (SHAP = −0.015) (Figure 4C).

**Figure 5:**
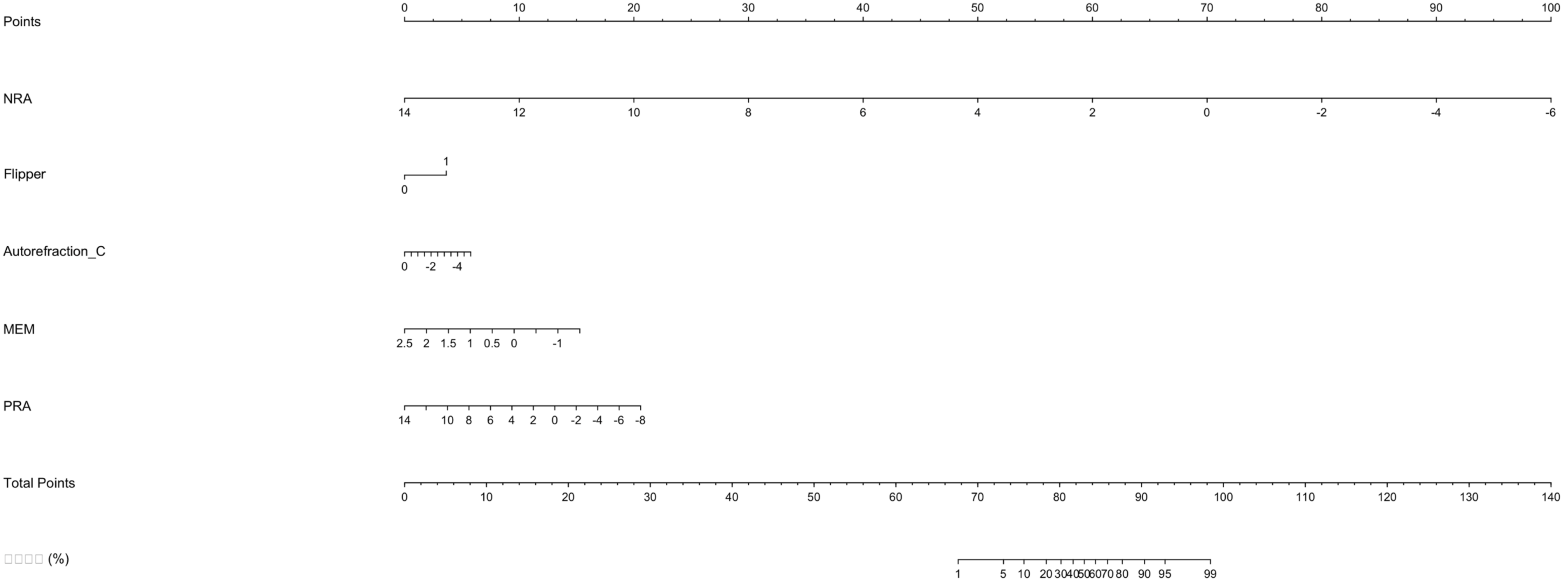
Nomogram for predicting DSE > 0.25 D in children and adolescents.

A nomogram was constructed based on the logistic regression model to convert individual clinical characteristics into a total score for estimating the probability of DSE > 0.25 D, thereby providing an intuitive and clinically applicable tool for individualized risk assessment (Figure 5).

### 3.5 Sensitivity analyses

First, generalized estimating equations (GEE) with participant-level clustering demonstrated results consistent with the primary logistic regression analysis. After accounting for within-subject inter-eye correlation, MEM (OR = 0.276, *p* = 0.006), NRA (OR = 0.228, *p* < 0.001), PRA (OR = 0.700, *p* = 0.001), cylindrical diopter (OR = 0.709, *p* = 0.002), and accommodative facility (OR = 3.329, *p* < 0.001) remained significantly associated with DSE > 0.25 D (Supplementary Table 1). Second, a single-eye sensitivity analysis was conducted by randomly selecting one eye per participant. The logistic regression model maintained comparable predictive performance, with an AUC of 0.877 (95% CI: 0.790–0.964), accuracy of 81.7%, sensitivity of 79.5%, specificity of 85.7%, and F1-score of 0.849 (Supplementary Table 2).

### 3.6 Quantitative analysis of the relationship between pre- and post-cycloplegic astigmatism

A strong linear relationship was observed between pre- and post-cycloplegic cylindrical diopter, with correlation analysis demonstrating an extremely strong positive association between the two measurements (Pearson’s r = 0.918, *p* < 0.001). The linear regression model showed a high goodness of fit (R^2^ = 0.842), with the regression equation: post-cycloplegic astigmatism = −0.066 + 0.874 × pre-cycloplegic astigmatism (*β* = 0.874, *p* < 0.001), from which the change between pre- and post-cycloplegic astigmatism was derived as: change = −0.066 − 0.126 × pre-cycloplegic astigmatism. Example calculations indicated that in patients with mild astigmatism (−1.00 D), the expected change was +0.06 D, whereas in those with high astigmatism (−4.00 D), the change could reach +0.44 D, confirming that more negative pre-cycloplegic astigmatism was associated with a larger cycloplegia-induced change.

## 4 Discussion

Accurate assessment of refractive status in children is fundamental for appropriate refractive correction and effective myopia control strategies. Because children have strong and unstable accommodation, non-cycloplegic refraction often deviates from cycloplegic refraction, leading to clinically meaningful errors. Large discrepancies between pre- and post-cycloplegic refraction may result in inappropriate prescriptions, inaccurate myopia risk stratification, and impaired visual development (3, 25, 26). Although cycloplegic refraction is widely regarded as the gold standard, its routine use is limited by photophobia, near-vision blur, allergic reactions, reduced patient compliance, and increased clinical workload. Therefore, identifying children at high risk of clinically significant DSE (>0.25 D) before cycloplegia would help optimize clinical decision-making and reduce unnecessary cycloplegic examinations.

In this retrospective study, eighteen non-cycloplegic variables, including demographic characteristics, anterior segment parameters, accommodative function, and binocular vision indices, were collected to construct machine-learning models for predicting whether the absolute DSE exceeded 0.25 D. Among the five algorithms tested, LR demonstrated consistently superior and stable performance with good interpretability. MEM, accommodative facility, NRA, PRA, and cylindrical diopter were identified as the final predictors, highlighting the central role of accommodative behavior in cycloplegia-induced refractive changes. The transparency of LR combined with SHAP-based interpretability supports its clinical applicability.

The LR model achieved an AUC of 0.871 (95% CI: 0.798–0.944) and an accuracy of 0.809, indicating good discriminative performance. Previous refractive prediction studies have mainly followed two approaches: deep learning based on fundus images and machine-learning models based on non-cycloplegic clinical parameters. However, their prediction errors remain relatively large. For example, Varadarajan et al. reported a best mean absolute error (MAE) of 0.56 D using conventional fundus photographs, while Yang et al. reported an MAE of approximately 1.75 D in highly myopic eyes using ultra-widefield images (19–21), which is insufficient for precise refractive assessment. By shifting the focus from predicting absolute refractive error to identifying individuals at risk of clinically meaningful cycloplegic change, the proposed model of this study achieved higher AUC and accuracy than those reported by Du et al. (AUC 0.833, accuracy ~77%) (22–24), further emphasizing the importance of accommodative-related functional indicators in determining the need for cycloplegia.

Notably, among the five variables ultimately included in the model, MEM, NRA, PRA, and accommodative facility are all closely related to accommodative function. This suggests that errors in non-cycloplegic refraction may primarily arise from instability of the accommodative system in children, rather than solely from refractive structural parameters. Compared with indirect indicators such as age and AL, functional accommodative parameters can more directly reflect children’s accommodative behavior under non-cycloplegic conditions, and therefore demonstrate higher predictive value in the model. From a physiological perspective, the proposed model can essentially be interpreted as an “accommodative function–based framework for evaluating the reliability of non-cycloplegic refraction,” further supporting the central role of accommodative function in pediatric non-cycloplegic refraction. On this basis, to further explain the contribution of individual variables, SHAP analysis was performed to visually and quantitatively interpret the internal mechanisms of the model. The SHAP summary plot showed that accommodative facility (failure to pass the plus-lens flipper test) had a strong positive contribution to DSE > 0.25 D, whereas MEM, NRA, PRA, and cylindrical diopter contributed negatively. Reduced accommodative facility indicates impaired relaxation or latent accommodative spasm, both of which can exaggerate myopic shift under non-cycloplegic conditions. Similarly, a lower NRA reflects reduced ability to relax accommodation and is associated with accommodative dysfunction. PRA, representing accommodative contraction ability, showed an inverse relationship: lower PRA values imply stronger accommodative tone and a higher likelihood of accommodative spasm (27–31), thereby increasing DSE. In contrast, MEM showed a negative contribution; higher MEM values indicate accommodative lag and reduced ciliary muscle contraction, limiting the magnitude of cycloplegia-induced refractive change, consistent with previous findings of a negative correlation between accommodative lag and DSE (32–37).

Cylindrical diopter showed a significant negative contribution to predicting DSE > 0.25 D, with a stronger impact as its absolute value increased. In this study, the relationship between pre- and post-cycloplegic astigmatism was quantified, and a strong linear correlation was observed (r = 0.918, *p* < 0.001), with the change in astigmatism described by the equation: ΔAstigmatism = −0.066 − 0.126 × pre-cycloplegic astigmatism. This suggests that mild astigmatism (−1.00 D) results in a minimal 0.06 D change, while high astigmatism (−4.00 D) leads to a change of up to 0.44 D. Higher-order aberrations, particularly coma, are positively correlated with corneal astigmatism (38–40) and both degrade retinal image quality (41, 42). In eyes with high astigmatism, this degradation increases accommodative effort during the refraction test, resulting in an overestimation of refractive error under non-cycloplegic conditions and larger pre-to-post cycloplegic differences. The quantitative findings of this study support this physiological mechanism.

Through SHAP analysis, the machine-learning model evolves from a black-box predictor into a clinically interpretable decision-support system. SHAP quantifies the positive and negative contributions of accommodative and refractive variables at the individual level, while the nomogram translates these weights into an intuitive visual scoring system. Together, they provide a transparent and operational framework for deciding whether cycloplegic refraction is necessary.

Although bilateral eye data were included in the primary analysis, additional sensitivity analyses using both GEE clustered regression and randomly selected single-eye data demonstrated materially consistent findings. The major predictors retained similar effect directions and statistical significance after accounting for within-subject inter-eye correlation, and the predictive performance of the logistic regression model remained stable in the single-eye analysis. These findings suggest that inclusion of bilateral eye data did not substantially bias the observed associations or model performance. Similarly, although thresholds of 0.50 D or 1.00 D are frequently used to define clinically meaningful refractive change in optometric practice, the present study selected a stricter threshold of 0.25 D because the primary aim was to assess agreement between non-cycloplegic and cycloplegic refraction rather than prescription-altering refractive change itself. Importantly, sensitivity analyses using alternative thresholds demonstrated relatively stable model performance, suggesting that the predictive framework is not highly dependent on a single cutoff definition and may generalize across different levels of refractive agreement.

Several limitations should be acknowledged. First, within a narrow threshold range of 0.67–0.72, the LR model provided a slightly lower net benefit than the “treat-all” strategy. However, because a probability threshold of 0.5 was used for classification, individuals within this range were still correctly classified as having DSE > 0.25 D, although their risk was underestimated. Second, this single-center retrospective design may limit generalizability, as regional, educational, and behavioral differences influence refractive and accommodative characteristics. In addition, the independent test cohort was relatively limited in size, which may further restrict the robustness and broader applicability of the model. Although internal validation and multiple sensitivity analyses demonstrated relatively stable model performance, external validation in larger multicenter populations is still required before broader clinical implementation. Third, MEM measurements are examiner-dependent and subject to variability; incorporating automated open-field autorefractors could improve reproducibility (43, 44). Finally, unmeasured behavioral factors such as reading distance, outdoor time, and near-work intensity may also influence accommodation and DSE and should be included in future studies.

## 5 Conclusion

This study developed a machine-learning model based on routine non-cycloplegic clinical parameters to predict whether the absolute DSE between pre- and post-cycloplegic refraction exceeds 0.25 D in children and adolescents. Logistic regression achieved the optimal balance between performance and interpretability. MEM, NRA, PRA, cylindrical power, and accommodative facility were identified as key predictors. This model provides a practical and clinically applicable decision-support tool for identifying individuals who truly require cycloplegic refraction, thereby reducing unnecessary cycloplegia and improving clinical efficiency. Further multicenter validation and incorporation of objective accommodative measurements may enhance its generalizability and clinical value.

## Data Availability

The raw data supporting the conclusions of this article will be made available by the authors, without undue reservation.
