# Supplementary material for: Assessing the reliability of non-cycloplegic refraction in children: a machine learning approach based on non-cycloplegic parameters
Source: Front Public Health. 2026 Jun 29;14:1822514. doi: 10.3389/fpubh.2026.1822514 (PMC13357664; doi:10.3389/fpubh.2026.1822514)
Supplement: Supplementary file 1 [file Data_Sheet_1.PDF]

# *Supplementary Material*

## 1. Supplementary Tables

**Supplementary Table 1 GEE clustered sensitivity analysis for the final logistic regression model**

| Variable | OR | 95%CI | P |
|---|---|---|---|
| MEM | 0.276 | 0.110–0.693 | 0.006 |
| NRA | 0.228 | 0.112–0.464 | <0.001 |
| PRA | 0.7 | 0.564–0.869 | 0.001 |
| Autorefraction_C | 0.709 | 0.570–0.883 | 0.002 |
| Flipper | 3.329 | 2.034–5.448 | <0.001 |

**Supplementary Table 2 Comparison of primary and single-eye sensitivity analyses**

| Metric | Primary analysis (547 eyes) | Single-eye sensitivity analysis (300 eyes) |
|---|---|---|
| AUC | 0.871 | 0.877 |
| 95% CI | （0.798，0.944） | 0.790–0.964 |
| Accuracy | 0.809 | 81.70% |
| Sensitivity | 0.771 | 79.50% |
| Specificity | 0.827 | 85.70% |
| F1-score | 0.720 | 0.849 |

**Supplementary Table 3 Sensitivity analysis of the final logistic regression model using alternative DSE thresholds**

| Metric | DSE > 0.25 D | DSE ≥0.50 D | DSE ≥ 0.75 D |
|---|---|---|---|
| AUC | 0.871 | 0.835 | 0.862 |
| 95% CI | 0.798–0.944 | 0.758–0.911 | 0.792–0.932 |
| Accuracy | 0.809 | 0.809 | 0.755 |
| Sensitivity | 0.771 | 0.833 | 0.833 |
| Specificity | 0.827 | 0.786 | 0.725 |
| F1-score | 0.72 | 0.811 | 0.649 |

## 2. Supplementary Figure

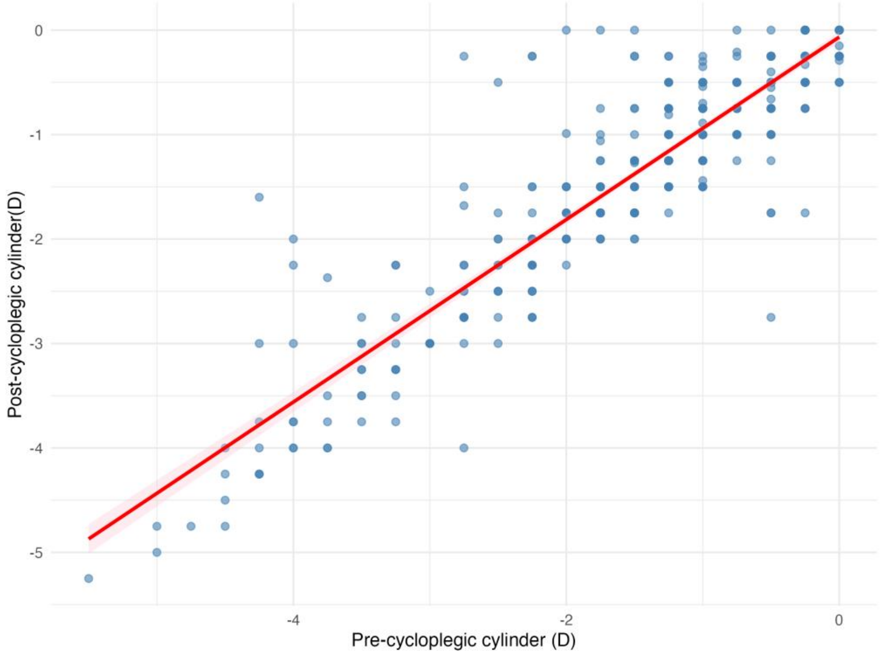


**Supplementary Figure 1.** Linear correlation between pre- and post-cycloplegic cylinder values.

Blue dots represent individual data points, the red line indicates the linear regression fit, and the shaded area represents the 95% confidence interval.
